# Diversity and composition of vegetation and soil seed banks after sand dune restoration by oil mulching and plantations

**DOI:** 10.1038/s41598-024-83095-y

**Published:** 2025-01-25

**Authors:** Banafsheh Jalilian, Noredin Rostami, Mehdi Heydari, Mehrdad Kohzadian, Orsolya Valkó, Reza Omidipour

**Affiliations:** 1 Department of Range and Watershed Management, Faculty of Agriculture, Ilam University, Ilam, Iran; 2 Department of Forest Science, Faculty of Agriculture, Ilam University, Ilam, Iran; 3 Ilam Natural Resources Office, Ilam, Iran; 4 ‘Lendület’ Seed Ecology Research Group, Institute of Ecology and Botany, HUN-REN Centre for Ecological Research, Vácrátót, Hungary

**Keywords:** Arid environment, oil mulching, restoration practices, soil seed bank, tree planting, Environmental sciences, Restoration ecology

## Abstract

Soil seed bank (SSB) is valuable reserves of seeds hidden in the soil and are especially important for the preservation and establishment of vegetation under adverse environmental conditions. However, there is a lack of knowledge on the effects of restoration measures on SSB, especially in arid ecosystems. Here, we assess the impacts of oil mulching (1 and 3 years after mulching) and plantations (15-year-old) on the diversity and composition of SSB and aboveground vegetation (AGV) in comparison with those in non-restored areas (i.e., control). Based on the results, species richness was poor in the studied area (36 species belonging to 16 families), with a lower contribution of SSB than of AGV (11 and 34 species, respectively). The largest number of exclusive species was observed in the planted treatment for both SSB and AGV (4 and 5 species, respectively), while the lowest was found in the 1-yr mulching treatment. The mean comparison of the diversity indices revealed that the highest values occurred in the plantation and 3-yr-mulching plots. The seed density in the plantation area was significantly greater (4145 ± 694 seeds/m^2^) than that in the other areas (3-yr-mulching > 1-yr-mulching > control treatments (145 ± 53, 65 ± 28, and 43 ± 22, respectively). The results of the DCA showed that the plantation treatment was completely separated from the other treatments in terms of the plant composition of the AGV, and the treatment closest to that area was 3-yr mulching, which indicates the positive effect of time since mulching on plant composition. The results of this study suggest that there is a trade-off between the short-term and medium-term effects of oil mulching, such that in the early years, oil mulching has a negative effect on the AGV and SSB, but its positive effects increase with time since mulching. It can be concluded that mulching, along with afforestation, creates a favorable microclimate and improves the diversity and composition of AGV and SSB.

**Supplementary Information:**

The online version contains supplementary material available at 10.1038/s41598-024-83095-y.

## Introduction

Arid and semiarid areas cover a large part of the Earth’s terrestrial surface, and more than a quarter of the world’s land is subjected to desertification^1,2^. Together with climate change and water shortages, desertification is considered a major global challenge in the 21st century^3^ and will lead to the loss of high-quality agricultural lands and the destruction of natural ecosystems, especially in arid and semiarid regions^4^. For this reason, it is very important to increase the understanding and awareness of methods for managing and preventing desertification, along with the use of appropriate methods of soil and vegetation modification and restoration in desert areas.

Oil mulching is one of the most important practices in the field of combating wind erosion and restoring vegetation in desert areas^5–8^. In general, the purpose of using oil mulch in quicksand stabilization activities is to increase the stability of the soil surface against wind erosion to create a suitable substrate for vegetation establishment activities such as planting, seeding, and establishment from vegetative organs^9,10^. However, in the early years, the use of oil mulch may have a negative effect on living organisms and plants^11^. Therefore, the use of oil mulch to stabilise sand dunes is still a challenge for land managers because of its negative and positive effects. Previous research has shown that oil mulching facilitates plant establishment and growth conditions^11^, increases the fertility and productivity of soil and positively affects vegetation establishment^12^. For example, a study^13^ reported a threefold increase in germination capacity of *Haloxylon persicum* in oil-mulched areas than in untreated areas in the central part of Iran. Another study^14^ also reported that oil mulching can improve seed germination. In another research, the planting of shrub seedlings under oil mulch was considered for stabilizing mobile sands. The results indicated that oil mulch and *Haloxylon* planting caused a significant decline in the percentage of total canopy cover of *Astragalus squarrosus* and density of *Astragalus squarrosus* and *Convolvulus hamadae*, but these practices increased the living aerial foliage volume of *A. squarrosus* and *C. hamadae*^15^. However, there are several negative effects of oil mulching on micro/macro-organisms and their shelters, soil properties and pioneer species that are adapted to unstable conditions^16–18^. Results of a study^11^ also reported the negative effects of oil mulching on species evenness and Shannon and Simpson diversity indices. The effects of oil mulching on aboveground vegetation (AGV) properties are well established; however, no studies have investigated the effects of oil mulching on the soil seed bank (SSB).

The SSB is an important component of plant communities^19,20^ and can play a key role in preserving and restoring degraded areas^21^. It includes a collection of germinable seeds in the soil that can drive the structure and dynamics of the AGV, with major effects on the temporal and spatial distribution of communities^22^.

Generally, the stability and resilience of sand dune habitats directly and indirectly depend on vegetation and SSB^23–25^. In fact, knowledge about the structure and dynamics of the SSB is very important for the management and restoration of the vegetation cover of degraded areas^24,25^. For example, by comparing AGV and SSB, it is possible to identify plants with persistent seed banks and support their use in restoring degraded areas^26^. To evaluate the restoration potential at particular sites, it is necessary to consider the density, diversity and distribution of the SSB and the similarity of the AGV and the SSB^27^.

Although many studies have been conducted in the SSB field in various climatic regions, arid desert areas have received considerably less attention (e.g^24,28^). , . Seed banks in desert areas usually consist of small seeds without specialized dispersal syndromes^29,30^. Under the right conditions, plant seeds can remain dormant until germination conditions are met in the soil for a certain period (from a few months to a few decades) and form the persistent SSB of that region until specific environmental conditions trigger germination^28^. This duration of survival is influenced by a range of abiotic and biotic factors and the characteristics of the plant species found in the seed bank. To date, there is a lack of knowledge regarding the changes in the diversity and composition of SSB and AGV after oil mulching at different time points.

Iran is a country in the arid and semiarid belt of the world in West Asia, where the area of desert exceeds 34 million hectares (20.6% of the total area of Iran), and poor rangelands with less than 25% vegetation cover approximately 16 million hectares (20.6% of the total area of Iran). In this regard, a study^31^ reported that 68% of Iran shows a high to very high susceptibility to desertification, and another study^32^ reported an increasing trend of desertification in the central parts of the country. Consequently, research on oil-mulch effects on AGV and SSB is highly important. In addition, information on the AGV and SSB dynamics along a chronosequence of oil-mulch application is necessary for informing decision-makers about the long-term feasibility of oil-mulching, its effects on AGV and SSB and the duration of its effects. To address these knowledge gaps, the current study aimed to investigate diversity and composition of vegetation and soil seed banks after sand dune restoration by oil mulching and plantations. Our aim was to answer the following questions:

1. Do the changes in the diversity of AGV and SSB have similar patterns with time since oil-mulching?
2. Does the number of exclusive species in AGV and SSB increase with time since oil-mulching?
3. Does the composition of AGV and SSB vary among different stages of secondary succession after oil mulching?

## Materials and methods

### Study area

**Fig. 1:**
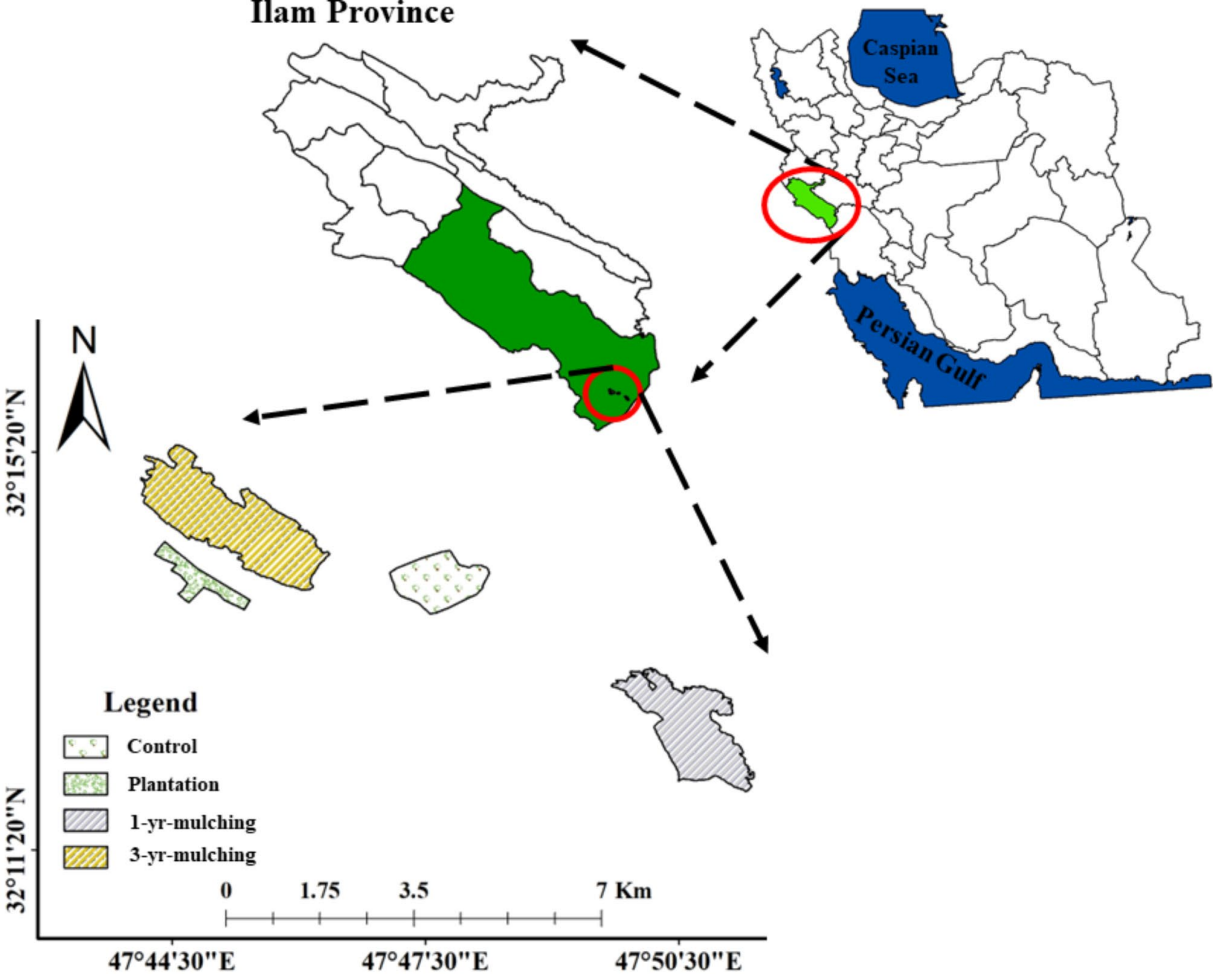
The location of the study areas in the southern part of the Ilam Province, western Iran.

The study area is located on the Abu Ghovir Plain, a part of Dasht-e-Abbas city in Dehloran County, Ilam Province, Iran (47°44ʹ to 47°50ʹ E and 32°11ʹ to 32°16ʹ N, Fig. 1). The average rainfall and mean annual temperature of the region are 210 mm and 26.2 °C, respectively^11^. The climate of the region is in the mid-warm desert class according to the Emberger method and in the hot dry class according to the De Martonne method^33,34^. Sand dunes and poor rangelands (1018.6 and 1307 ha, respectively) are the main land uses in Dasht-e-Abbas, followed by irrigated crops (182.9 ha) and rain-fed crops (149.6 ha). The region’s soil needs stabilisation, both because of its desert nature and loose sands, and because of the people’s dependence on the region’s poor rangelands, as well as the change in land use to agriculture.

To stabilize the soil of this area and restore the vegetation, the following measures were taken by the Ilam Department of Natural Resources:

- Oil mulching in 2017 (3 year of mulching).
- Oil mulching in 2019 (1 year of mulching).
- Plantation with *Prosopis juliflora* seedlings in 2005 (15-year-old).
- Control area (no mulching, no plantation).

These study sites were located in the same physiographic conditions (flat; slope < 8%) and the elevation ranged from 60 to 220 m above sea level) and were independent from each other spatially.

### Seed bank sampling method

SSB sampling was performed in early October 2020, after the end of the growing season and seed ripening period and before autumn germination. Sampling from the SSB was performed using a sampling device with dimensions of 20 × 20 cm at a depth of 0–5 cm. In this way, 60 randomly placed soil samples (15 per treatment type) were collected. Then, the soil samples were put into labeled plastic bags, transferred to the laboratory for cold storage and kept at a temperature of 3 to 4 °C for three months. After artificial cooling, the samples were transferred to the greenhouse environment at the Agriculture Faculty of Ilam University (temperature conditions of 18 to 25 °C and sufficient humidity), and the seed bank study was conducted based on the seedling emergence method by cultivation in a greenhouse^35^. During the growing season, we visited the sampling area again and estimated the percentage of cover of the AGV corresponding to the SSB samples in one square meter sample plots.

### Greenhouse cultivation

The SSB samples were cultivated in a greenhouse environment with suitable temperature conditions of 18 to 25 degrees Celsius and sufficient humidity inside trays (with dimensions of 14 × 14 × 3 cm) in a suitable substrate. Inside each tray, soil samples were spread on a thin layer of sterile sand (thickness of 3 cm) so that their thickness was not more than 2 cm so that all the seeds were exposed to light and air and had a high chance of germination^36^. The trays were randomly placed in rows under a natural light regime and kept moist. To detect possible contamination by airborne seed rain, for every 10 trays, one tray containing only sterile sand was placed among the sample trays as a control. After growing in the greenhouse, the emerging plants were counted, identified and finally removed from the trays at regular intervals until no more plants germinated. After one month, when no more seeds sprouted from inside the trays, the irrigation was stopped for two weeks, and after a surface scratch in the soil of the trays, the irrigation started again, and the counting started until no more seedlings emerged^36^.

### Vegetation sampling

To compare the composition and diversity of vegetation between the studied sites in a split plot design (1-yr-mulching, 3-yr-mulching, planted and control sites), a similar surface located in the center of each stand was studied by a systematic random method. To increase the accuracy, taking into account the characteristics of the representative stands, three repetitions were performed for each site and at the same location where the seed bank was sampled. In each repetition, two perpendicular transects 20 m in length were laid out with a random start. The sampling of vegetation at each site was performed using 15 quadrats (sample pieces) of 1 square meter (five quadrats along each transect and one at the intersection of two transects) along 3 transects of 100 m (3 transects at each site). At the end of April, 2022 of the vegetation was recorded in each quadrat according to the Braun-Blanquet approach^37^. The identification of plant species was performed using the flora of Ilam^38^. The life forms of all identified species were determined based on Raunkiaer’s classification^39^.

### Statistical analyses

To quantify the different aspects of species diversity, we expressed species/seed richness (numbers of species/seeds per plot), evenness (e^H/S^), and Shannon and Simpson indices^40^. In addition, seed density (number of seeds/m^2^) and AGV cover were measured. On ther other hand, we used rarefaction and extrapolation curve based based on Hill’s diversity index at three orders of q-value (i.e., q = 0, q = 1 and q = 2, corresponding to species richness, Shannon and Simpson diversity indices, respectively). The aim of rarefaction is to standardise the uneven number of spamples, and extrapolation allowed prediction of real diversity, taking into account the estimation of species not covered by the sampling effort. Here, we used a coverage-based^41^ rarefaction/extrapolation for individual-based data (SSB) and abundance-based data (i.e., AGV). This statistical analysis was carried out using the packages “iNEXT”^42^ and “ggplot2”^43^. Before performing the statistical analysis, the normality of the average and the homogeneity of the variance of the vegetation diversity and SSB data were checked by using the Kolmogorov‒Smirnov and Levene’s tests, respectively. Due to the existence of several quadrats without plant species (especially in the SSB) or plots with only one species, all the investigated indices had nonnormal distributions. In such cases, the use of conventional statistical functions (such as logarithm and square root transformations) will not improve the normality of the data. We used a generalized linear mixed model (GLMM), which can analyze data with any type of non-normal distribution pattern. Due to the different formats of the investigated data, different distribution patterns were used in the GLMM tests. This analysis was performed using “lme4” and “car” packages in R. To investigate the changes in the species composition of SSB and AGV at the different sites (1-yr-mulching, 3-yr-mulching, control and planted sites), we used detrended correspondence analysis (DCA) based on the floristic composition of the samples as an indirect gradient analysis in the software PC-Ord Ver. 4.17.

## Results

### Vegetation composition

Overall, 36 plant species belonging to 32 genera and 16 families were observed (sum of the AGV and the SSB) in the study area. Among these species, 2 were exclusive to SSB (*Calendula persica* and *Medicago radiata*), 25 were exclusive to AGV, and 9 families were present in both SSB and AGV (Table 1).

In the AGV, there were a total of 34 species belonging to 30 genera and 16 families, with Asteraceae (8 species), Poaceae (7 species), and Fabaceae (5 species) containing the greatest number of species (Table 1, S1). Additionally, the largest genus in terms of the number of species was *Trifolium*(*T. lappaceum*, *T. tomentosum*, and *T. resupinatum*) (Table S1).

The SSB contained 11 plant species belonging to 11 genera and 9 families (Table 1). In terms of the number of species in the SSB, the largest family was *Poaceae*(*Stipa capensis*, *Lophochloa phleoides*, and *Bromus scoparius*) (Table S1).

**Table 1:** The number of plant families in the soil seed bank and aboveground vegetation.

Section	Number				
	Species	Family	Exclusive Species	Exclusive Family	Common Families
Soil Seed Bank	11	9	2	0	9
Aboveground vegetation	34	16	25	7	
Overall	36	16			

The investigation of the growth form type showed that 81.8% (9) of the SSB species were therophytes and 18.2% (2) were hemicryptophytes (Table S1). Similarly, in the AGV, 79.4% (27 species) of the species were therophytes and 20.6% (7 species) were hemicryptophytes (Table S1).

The results of the chorology investigation showed that 41.2% of the recorded species in the AGV related to the Iran-Turani- Sahara-Sindian vegetation zone, and 20.6% of the species belonged to the Iran-Mediterranean Turani vegetation zone. Additionally, 45.5% of the recorded SSB species belonged to the vegetation zone of the Iran–Turani– Sahara-Sindian region, 27.8% of the species belonged to the vegetation zone of the Iran–Turani–Mediterranean region, and other chorotypes were present at lower percentages in the region. (Table S1).

**Fig. 2:**
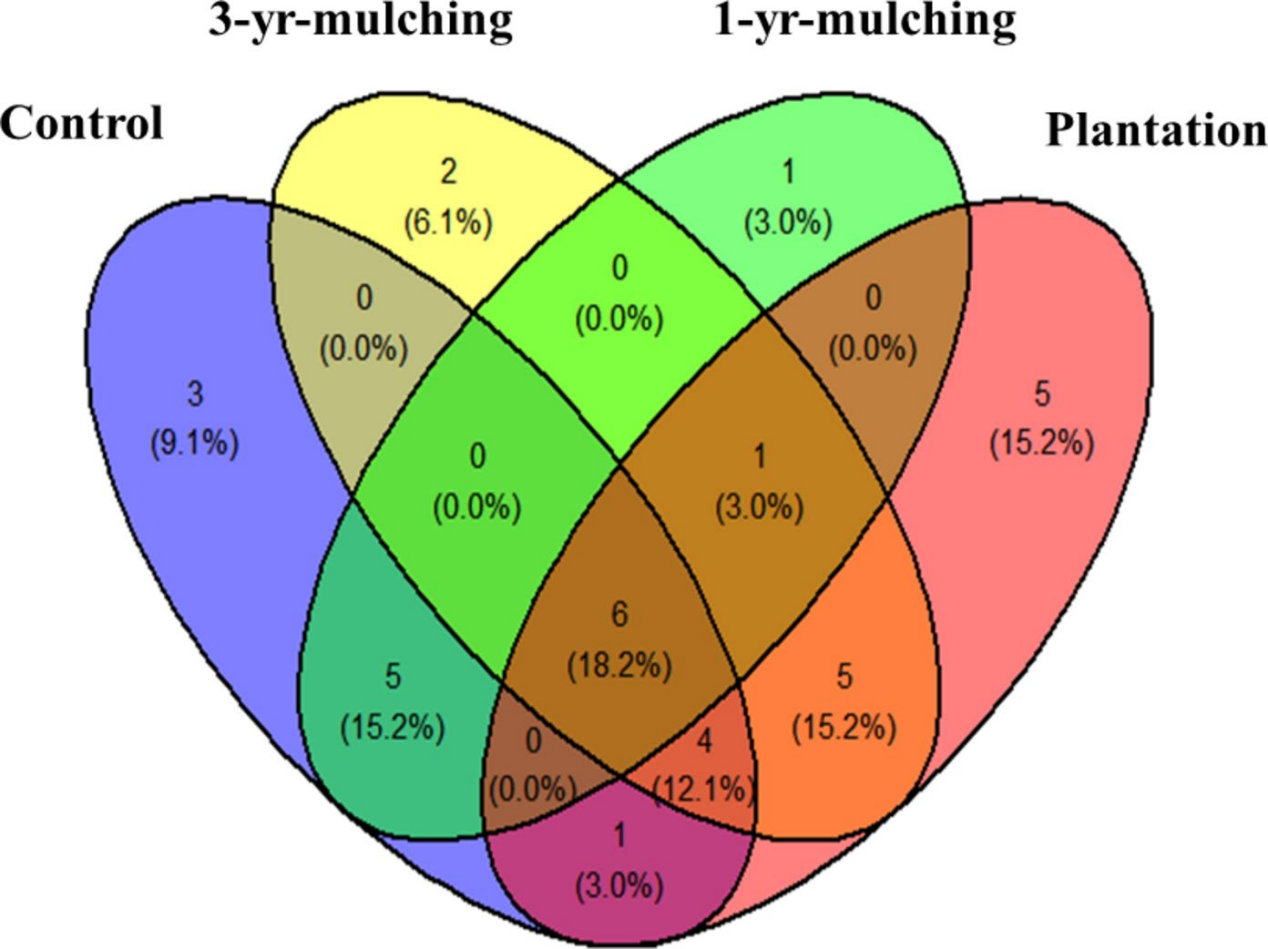
Venn diagram for common and exclusive plant species in aboveground vegetation. The numbers inside the parentheses indicate the percentage of all species, and the numbers outside the parentheses indicate the number of species in that state.

Investigating the plant composition of the AGV among the four treatments revealed six common species (*Trigonella anguinea*, *Trifolium lappaceum*, *Plantago amplexicaulis*, *Neurada procumbens*, *Cornulaca aucheri*, and *Asphodelus tenuifolius*), while the largest number of exclusive species was observed in the planted treatment (five species: *Trifolium resupinatum*, *Hordeum glaucum*, *Hedypnois rhagadioloides*, *Bromus scoparius*, and *Anagallis arvensis*) (Fig. 2 and Table S1). The greatest number of common species in the planted treatment was related to 3-yr-mulching with 5 species (*Taraxacum montanum*, *Malva parviflora*, *Heliotropium ramosissimum*, *Emex spinosus*, and *Stipa capensis*), followed by the control area with one species (*Bromus tectorum*), while 1-yr-mulching had no species in common with the 3-yr-mulching and planted treatments (Fig. 2 and Table S1).

**Fig. 3:**
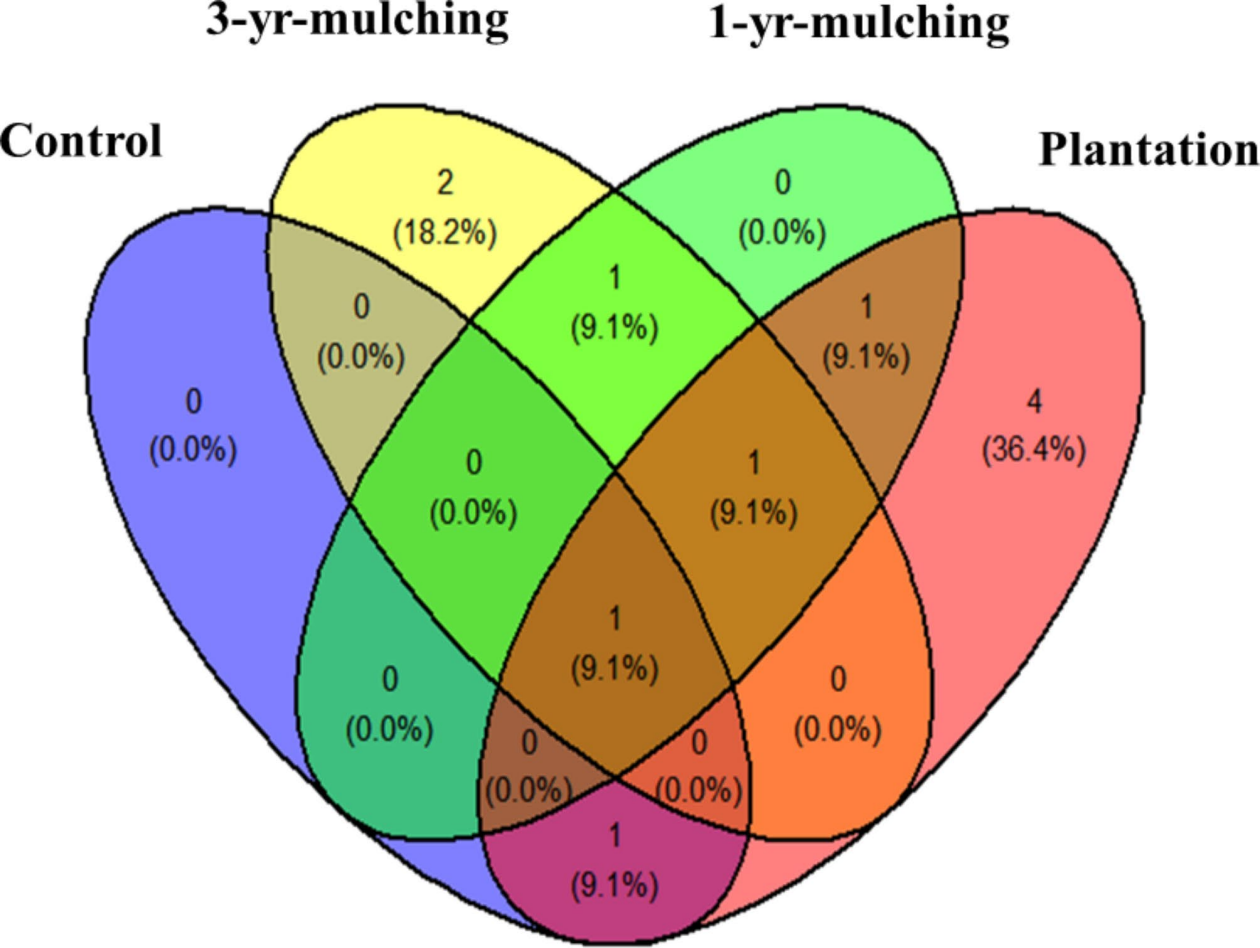
Venn diagram for common and exclusive plant species in the soil seed bank. The numbers inside the parentheses indicate the percentage of all species, and the numbers outside the parentheses indicate the number of species in that state.

Investigating the plant composition of the SSB showed that there was only one common species (*Plantago amplexicaulis*) among all treatments (Fig. 3 and Table S1). Additionally, the greatest number of exclusive species was related to the four planted species (*Stipa capensis*, *Calendula persica*, *Bromus scoparius*, and *Anagallis arvensis*), followed by the two 3-yr-mulching species (*Malva parviflora* and *Emex spinosus*), while there were no exclusive species in the 1-yr-mulching and control treatments (Fig. 3 and Table S1).

### Comparison of the diversity of aboveground vegetation and the soil seed bank

The GLMM results showed that there was a significant difference (*P* value < 0.05) between the diversity indices in the two groups of AGV and SSB among the different treatments, and only the species evenness of SSB was not significantly different between the treatments (*P* value = 0.779). Additionally, the two variables of vegetation cover and seed density exhibited significant differences among the different chronosequences after oil mulching (Table 2).

**Table 2:** GLMM analysis results for diversity indices of aboveground vegetation and the soil seed bank among different treatments after oil-mulch application.

Group	Indices	df	Chi Squared (X^2^)	*P* value
Aboveground vegetation	Vegetation cover	3	305.63	< 0.001
	Species richness	3	104.71	< 0.001
	Shannon index	3	81.137	< 0.001
	Simpson index	3	35.95	< 0.001
	Evenness index	3	90.002	< 0.001
Soil seed bank	Seed density	3	205.54	< 0.001
	Species richness	3	28.47	< 0.001
	Shannon index	3	16.19	0.001
	Simpson index	3	9.18	0.02
	Evenness index	3	1.009	0.779

**Fig. 4:**
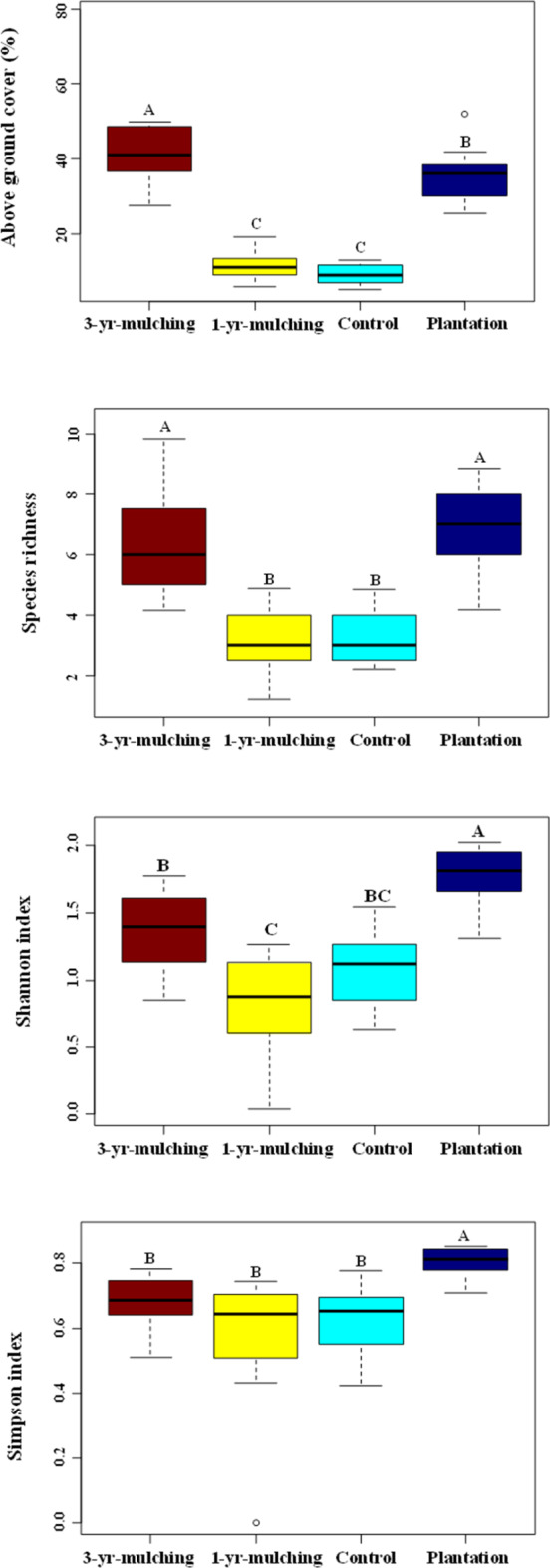
Means and standard errors of the total vegetation cover and different diversity indices of aboveground vegetation among the different treatments after oil mulching. Different lowercase letters indicate significant differences between treatments.

There was a similar pattern for vegetation cover and species richness in the AGV, and the highest values were related to 3-yr mulching, followed by the planted treatment, and the lowest values were found in the 1-yr mulching and control sites (Fig. 4).

Among the diversity indices (Shannon and Simpson indices), the highest values were associated with the planted and 3-yr-mulching treatments. The highest values of the evenness index were related to the control site, followed by the 1-yr-mulching, planted and 3-yr-mulching treatments (Fig. 3).

**Fig. 5:**
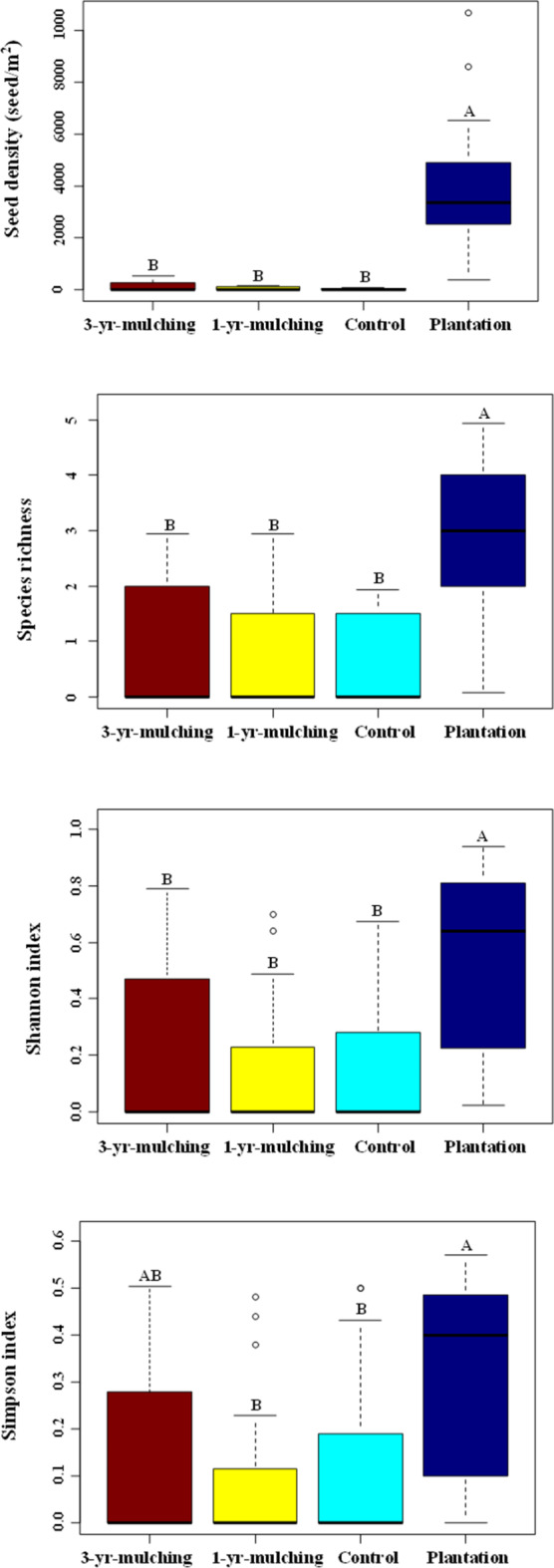
Means and standard errors of the seed density and different diversity indices of the soil seed bank among the different treatments after oil mulching. Different lowercase letters indicate significant differences between treatments.

Similar patterns were observed for the diversity indices in the SSB (species richness, Shannon and Simpson indices), and the highest values were found at the planted site (Fig. 5). Similarly, there was the same pattern for seed density among the different treatments after oil mulching, in the order of planted > 3-yr-mulching > 1-yr-mulching > control treatments (Fig. 5).

**Fig. 6:**
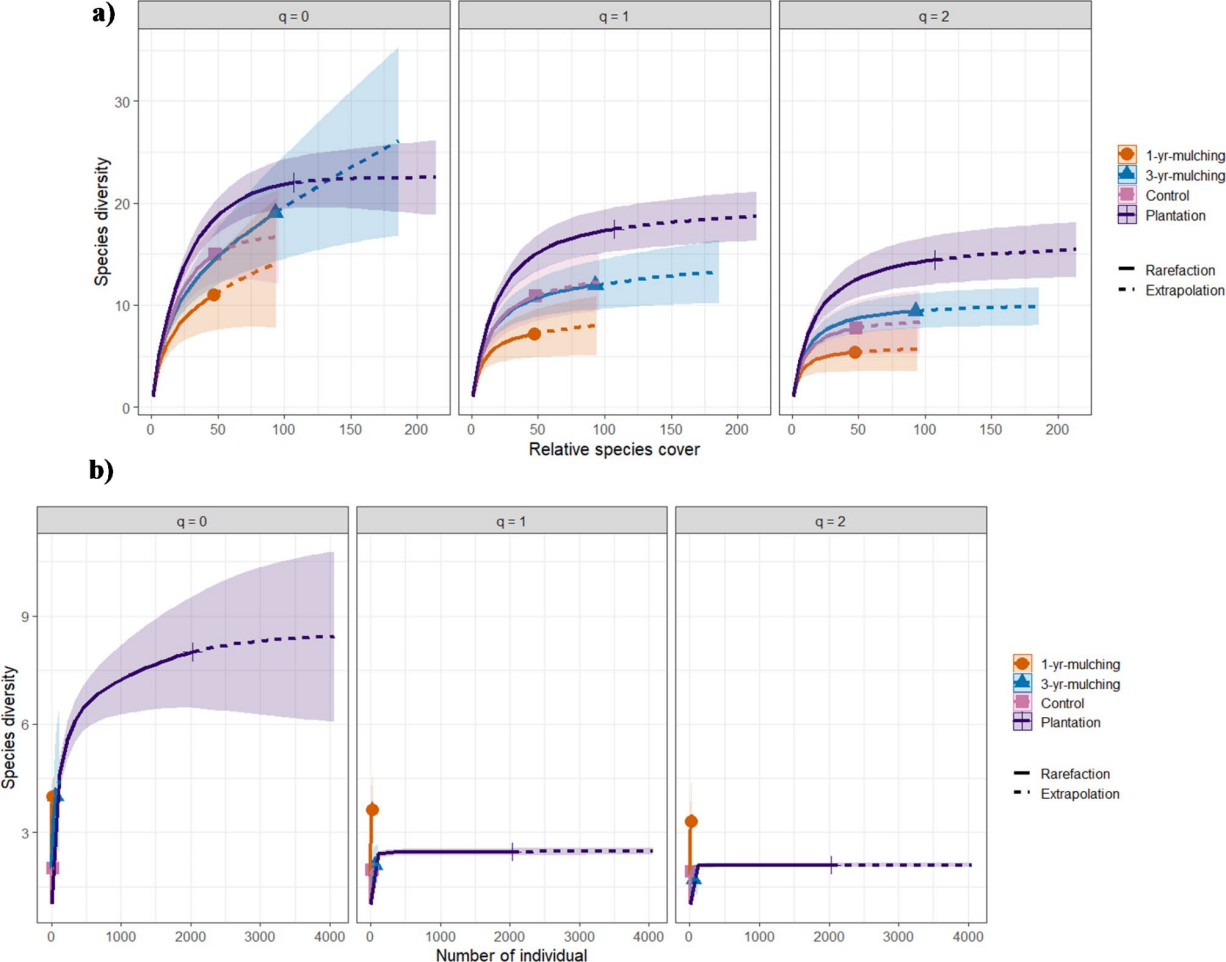
Cover-based rarefaction/extrapolation biodiversity curves as functions of the relative cover (AGV) and number of seedling (SSB), based on Hill’s index at three levels q = 0, q = 1 and q = 2 that corresponding to species richness, Shannon diversity and Simpson diversity indices, respectively for AGV (**a**) and SSB (**b**). The solid and dashed lines are the rarefaction and extrapolation curves, respectively. The shaded area indicates 95% confidence intervals obtained using the bootstrap technique based on 1000 replicates.

The study of the diversity of SSB and AGV using the Hill index at three levels q = 0, q = 1 and q = 2, corresponding to the species richness, Simpson’s diversity and Shannon’s diversity indices, respectively, showed that for both AGV and SSB, the highest and lowest values of diversity at all levels considered were found in the plantation and 1-yr-mulching respectively (Fig. 6).

### Detrended correspondence analysis (DCA)

**Fig. 7:**
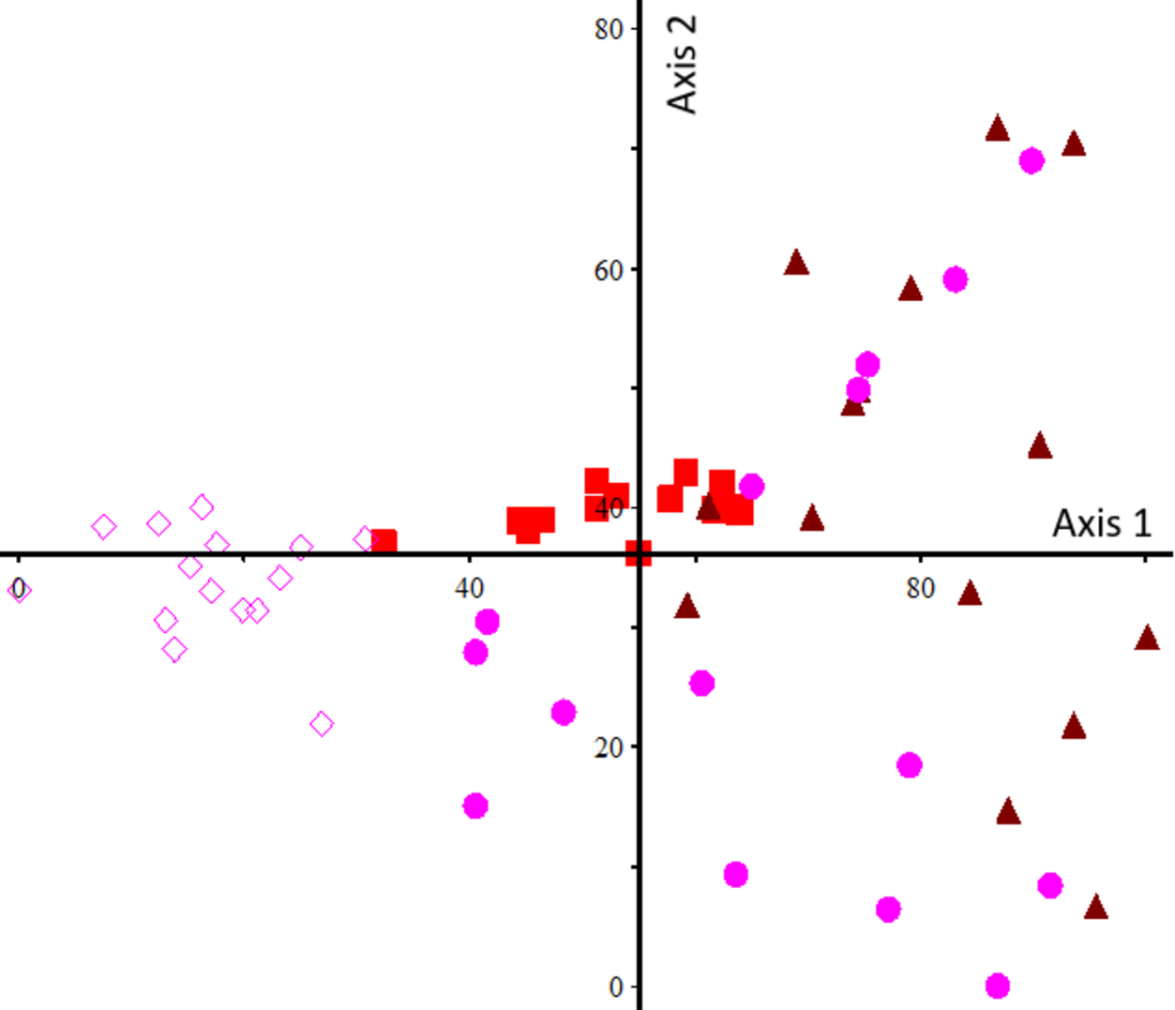
Detrended correspondence analysis (DCA) of the aboveground vegetation; red filled rectangle: 3-yr-mulching, brown filled triangle: 1-yr-mulching, open pink diamond: Plantation, filled pink circle: Control.

The results of DCA analysis of the first and second axes, with eigenvalues of 0.627 and 0.401, respectively, explained the greatest percentage of the total changes in plant composition (Fig. 7). Based on this analysis, the planted treatment was completely separated from the other treatments in terms of plant composition and formed a group at the end of the negative direction of the first axis. Additionally, the 3-yr-mulching area was determined to have a plant composition similar to that of the planted treatment. Moreover, the 1-yr-mulching and control samples did not form distinct groups, indicating that their species compositions were highly similar (Fig. 7). The life form and geographical distribution of each of the plant species related to each of the ecological groups are listed in Tables S2 to S4.

## Discussion

The results showed that in all the treatments, especially in the plantations, the AGV had greater plant species richness than did the SSB. A greater richness of AGV than SSB has been reported in different studies^44^. This is because not all species have the ability to form an SSB^27,45^, and the seeds of some species need additional treatments for breaking dormancy^46,47^. There were nine exclusive families, such as Liliaceae, Convolvulaceae, Cruciferae and Geraniaceae, in the AGV, and several exclusive species, such as *Asphodelus tenuifolius*, *Artemisia sieberi*, *Convolvulus oxyphyllus*, *Diplotaxis harra*, *Emex spinosus*, *Neurada procumbens*, *Trifolium lappaceum* and *Trigonella anguinea*, which were only recorded in the AGV and were absent from the SSB. These results highlight that in the studied arid environment, the recovery of several native species needs to be aided by active restoration interventions, such as seeding or planting.

Among the treatments, the plantation and 3-yr mulching treatments had greater species richness than did the 1-yr mulching and control treatments. The tree species in the plantation treatment created a milder microclimate and improved the humidity, temperature and soil nutrients locally, which could have led to the increased germination capacity of the species and their establishment in the AGV, which enabled the regular build-up of SSB^48^. In line with our results, another study^49^ reported that planting in dry regions by changing soil characteristics has increased the potential for plant restoration based on SSB.

Our results showed that sites with longer periods since oil-mulching had greater AGV cover and species richness than sites with shorter periods since oil-mulching. No significant differences were found between the seed bank density and richness between the two oil-mulching treatments, but there was a tendency for larger and more diverse seed banks with increasing time since oil-mulching. In desert and plain areas, soil stabilization with mulch can reduce soil temperature, buffer humidity fluctuations and prevent erosion^50^. In such conditions and with the passage of time since mulching, with the establishment of pioneer species from the SSB, effective and rapid growth and development of the AGV occur; therefore, the soil seed reserve is strengthened every year^51,52^. Both the SSB and the AGV of the study system were dominated by therophytes and hemicryptophytes, with a contribution of nearly 80%. These two life forms, especially therophytes, have been reported as the dominant life form in the flora of many arid and semiarid regions^53,54^. Therophytes are annual species with the ability to produce abundant and small seeds, which can strengthen the seed bank in arid conditions, in addition to their establishment in the AGV every year^55^. The results showed that some plants, such as *Astragalus asterias*, *Heliotropium ramosissimum*, *Taraxacum montanum*, and *Trifolium resupinatum*, were present exclusively in the AGV of the older mulching treatment and/or in the planted area. These results suggest that milder temperature and humidity conditions are needed for the emergence of these species.

Venn diagrams showing the similarity of the plant composition between the treatments by separating two sections of AGV and SSB showed that the degree of similarity between the treatments was low, and the greatest similarity was recorded in the AGV between the plantation and 3-yr-mulching treatments (5 species). The similarity of the AGV composition between the plantation and 3-yr-mulching treatments indicates that these treatments had similar positive effects on the restoration of vegetation. Mulching in the early years may have negative effects on vegetation^11^. Oil mulch in the first few years can cause temporary or permanent elimination of some species. For example, the results of the present study showed that mulching caused the elimination of plants such as *Chrozophora tinctoria*, *Stipagrostis plumosa*, and *Cymbolaena griffithii* from the vegetation composition, and these plants were still missing from the AGV in later successional stages. Therefore, in addition to mulching, additional measures such as seeding and planting are recommended for a more complete restoration of native species (see also^56^). The results showed that by increasing the age of mulching to three years, exclusive species were included in the composition of the seed bank, while no exclusive species were recorded in the control areas or in the early years of mulching. This result shows that the positive effects of mulching are expressed after at least three years (see also^57^). Mulching treatment improves soil conditions, reduces erosion, provides conditions for the establishment of pioneer plants, and increases the possibility of establishing new plants.

Investigating the diversity and richness of both the SSB and AGV showed that these indices were significantly greater in the 3-yr mulching and plantation treatments than in the 1-yr mulching and control mulch treatments. A previous study in this region^11^, also reported a positive effect of short-term oil-mulching on vegetation cover, plant litter and rangeland condition (i.e., rangeland condition score); while, diversity indices (Shannon and Simpson indices) 63%, and 71%, declined by applying oil-mulch (respectively). Therefore, it can be concluded that the early positive effects of mulching on sand dune stabilizing will continue and increase over time. For instance, with the increase of seed entry into the region and the emergence and establishment of perennial plants (i.e., nursing plant), the plant flora of the region will be enriched and the effects of these positive changes in improving the soil will also be enhanced^58–61^. However, for the evenness index, a completely different pattern was observed. Based on the results for both AGV and SSB, the evenness index was greater in the 1-yr-mulching and control treatments than in the other treatments. This result could be due to the limited number of plant species in these two treatments. In other words, in the control area, due to the unfavorable environmental conditions and in the 1-yr mulching period due to the negative effects of the oil mulch in the early years, the species pool was limited, which increased the evenness of the plant composition. With the passage of time since mulching and improvements in environmental conditions, certain species became dominant, and several subordinate species were added to the communities, which resulted in an increase in plant diversity and decreased evenness values. This difference in abundance will reduce the evenness of the plant composition in the AGV and SSB.

Examining the seed density in the SSB of the investigated treatments showed a large difference between the plantation and other treatments. This can be partly explained by the milder environmental conditions in this treatment (moisture, temperature and accumulation of nutrients due to the decomposition of dead leaves) and by the seed trap effect of the litter that prevents the movement of seeds^62,63^. In addition, the high diversity and abundance of plant species in the plantation treatment, especially for therophytes with abundant and small seeds, increased the seed density (400 seeds/m^2^).

Investigating the plant composition using the DCA indicated a clear distinction the plantation area from the other treatments. In other words, plantations caused a clear change in the plant composition of AGV. The plant composition of the plantation samples in the 3-yr-mulching treatment group was similar to that in the DCA group, which also indicates that these two treatments had greater similarities in terms of plant composition, while the 1-yr-mulching treatment group and the control group did not have homogeneous or distinct compositions. These results show that with increasing time since mulching, the plant community has stabilized and has obtained a stable and homogeneous plant composition, which will likely increase its stability against environmental changes and disturbances. Planting with native species can improve suitable environmental conditions and soil characteristics in the studied habitat, which will strengthen and clearly change the composition of the understory vegetation^49^. These results imply that it is possible to give an establishment advantage to native species of the region by creating the initial establishment conditions by the use of oil mulching. However, in the early years, the established plants will have very little resistance, and the need for auxiliary and protective measures is very high.

## Conclusion

The results of this study highlight the potential of different soil stabilization treatments for vegetation restoration in the studied arid ecosystems. By creating a milder microclimate, both the mulching and planting treatments increased the species richness of the vegetation and the seed bank. We found that the positive effect of mulching became evident only a few years after the application of the treatment, but not in one year after oil mulching, which highlights the necessity of long-term monitoring programs for a comprehensive evaluation of restoration success. We found that in the studied ecosystem, most of the native plant species did not possess persistent SSB, which highlights the importance of active propagule addition of the desired target species. Considering the negative effects of oil mulching one year after its application, it is necessary to carry out protective measures for native and planted species, especially in the first year of oil mulching.

## Electronic Supplementary Material

Below is the link to the electronic supplementary material.

Supplementary Material 1

## Data Availability

The data used to support the findings of this study are available from the corresponding author upon reasonable request.

## References

[1] D’Odorico, P., Bhattachan, A., Davis, K. F., Ravi, S. & Runyan, C. W. Global desertification: drivers and feedbacks. *Adv. Water Resour.* **51**, 326–344. 10.1016/j.advwatres.2012.01.013 (2013).

[2] Huang, J. et al. Global desertification vulnerability to climate change and human activities. *Land. Degrad. Dev.* **31**(11), 1380–1391. 10.1002/ldr.3556 (2020).

[3] Zare, S. et al. Effects of different mulches on soil and plants in the desert of Iran. *Arab. J. Geosci.* **15**(10), 1–14. 10.1007/s12517-022-10040-6 (2022).

[4] Anjum, S. A. et al. Desertification in Pakistan: causes, impacts and management. *J. Food Agric. Environ.* **8**, 1203–1208 (2010).

[5] Amiraslani, F. & Dragovich, D. Combating desertification in Iran over the last 50 years: an overview of changing approaches. *J. Environ. Manage.* **92**(1), 1–13. 10.1016/j.jenvman.2010.08.012 (2011). 20855149 10.1016/j.jenvman.2010.08.012

[6] Shojaei, S., Ardakani, M. A. H. & Sodaiezadeh, H. Optimization of parameters affecting organic mulch test to control erosion. *J. Environ. Manage.* **249**, 109414. 10.1016/j.jenvman.2019.109414 (2019). 31445368 10.1016/j.jenvman.2019.109414

[7] Emadodin, I., Reinsch, T. & Taube, F. Drought and desertification in Iran. *Hydrology* **6**(3), 66. 10.3390/hydrology6030066 (2019).

[8] Rabani, S., Ordookhani, K., Aref, F., Zare, M. & Sharafzadeh, S. Soil Physicochemical Properties and Caper (Capparis spinosa L.) Growth in Response to Various Mulches. *Commun. Soil Sci. Plant Anal.* **54**(1), 128–140. 10.1080/00103624.2022.2110259 (2023).

[9] Akbarnia, H. The evaluation of contaminated soil by petroleum mulch in combating desertification. *Desert* **14**(2), 127–132 (2009).

[10] Amiraslani, F., Dragovich, D. & Caiserman, A. *A long-term cost–benefit analysis of national anti-desertification plans in Iran* 141 (Desert, 2018).

[11] Rostami, N., Karimi, H., Tavakoli, M. & Omidipour, R. Short-term effect of oil‐mulch on vegetation dynamics; Integration of ecological and remote sensing‐based approaches. *Land. Degrad. Dev.* **33**(2), 235–245. 10.1002/ldr.4140 (2022).

[12] Shojaei, S., Ardakani, M. A. H., Sodaiezadeh, H., Jafari, M. & Afzali, S. F. New laboratory techniques (novel) in making organic-mineral mulch to control wind and water erosion and its use in global scale. *Spat. Inform. Res.* **29**(1), 97–107. 10.1007/s41324-020-00335-9 (2021).

[13] Jafarian, V. The effect of petroleum mulching on seed germination of desert plants in Iran. International Symposium on Drylands Ecology and Human Security, Dubai, Abstract 2006/154 (2006).

[14] Farahpour, M., Ghayour, F. A., Sherbaf, H. & Yousefizadeh, A. Comparison of water absorbent and nonoil mulch with oil mulch on seed germination and sand dune stabilization. *Iran. J. Range Desert Res.* **12**, 121–134. 10.22092/ijrdr.2019.119935 (2005).

[15] Gholami Tabasi, J., Jafary, M., Azarnivand, H. & Sarparast, M. Vegetation and soil properties of a sandy desert affected by shrub (*Haloxylon aphyllum*) plantation and oil mulching. *Environ. Resour. Res.* **2**(1), 67–76. 10.22069/IJERR.2014.1681 (2014).

[16] Mario, O. S., Oscar, L. A., Octavio, P. Z. & Felipe, D. S. Effect of transparent mulch, floating row covers and oil sprays on insect populations, virus diseases and yield of cantaloup. *Biol. Agric. Hortic.* **10**(4), 229–234. 10.1080/01448765.1994.9754675 (1994).

[17] Yan, S. & Liu, Z. Effects of dune stabilization on the plant diversity of interdune wetlands in northeastern Inner Mongolia, China. *Land. Degrad. Dev.* **21**(1), 40–47. 10.1002/ldr.966 (2010).

[18] Rudolf, K., Hennings, N., Dippold, M. A., Edison, E. & Wollni, M. Improving economic and environmental outcomes in oil palm smallholdings: The relationship between mulching, soil properties and yields. *Agric. Syst.* **193**, 103242. 10.1016/j.agsy.2021.103242 (2021).

[19] Baskin, C. C. & Baskin, J. M. *Germination Ecology of Seeds in the Persistent Seed Bank. Ecology, Biogeography, and Evolution of Dormancy and Germination* 133–180 (Academic, 2014). 10.1016/B978-0-12-416677-6.00007-X

[20] Yang, X. et al. Global patterns of potential future plant diversity hidden in soil seed banks. *Nat. Commun.* **12**(1), 7023. 10.1038/s41467-021-27379-1 (2021). 34857747 10.1038/s41467-021-27379-1 PMC8639999

[21] Wang, Y. et al. Sand dune stabilization changes the vegetation characteristics and soil seed bank and their correlations with environmental factors. *Sci. Total Environ.* **648**, 500–507. 10.1016/j.scitotenv.2018.08.093 (2019). 30121529 10.1016/j.scitotenv.2018.08.093

[22] Fenner, M. & Thompson, K. *The ecology of seed banks* 250 (Cambridge University Press, 2005). 10.1017/CBO9780511614101

[23] Liu, B., Liu, Z., Wang, L. & Wang, Z. Responses of rhizomatous grass Phragmites communis to wind erosion: effects on biomass allocation. *Plant. soil.* **380**, 389–398. 10.1007/s11104-014-2104-y (2014).

[24] Qian, J., Liu, Z., Hatier, J. H. B. & Liu, B. The vertical distribution of soil seed bank and its restoration implication in an active sand dune of northeastern Inner Mongolia, China. *Land. Degrad. Dev.* **27**(2), 305–315. 10.1002/ldr.2428 (2016).

[25] Tóth, Á. et al. Vertical distribution of soil seed bank and the ecological importance of deeply buried seeds in alkaline grasslands. *PeerJ* **10**, e13226. 10.7717/peerj.13226 (2022). 35402097 10.7717/peerj.13226 PMC8992659

[26] Luo, C., Guo, X., Feng, C. & Xiao, C. Soil seed bank responses to anthropogenic disturbances and its vegetation restoration potential in the arid mining area. *Ecol. Ind.* **154**, 110549. 10.1016/j.ecolind.2023.110549 (2023).

[27] Bossuyt, B. & Honnay, O. Can the seed bank be used for ecological restoration? An overview of seed bank characteristics in European communities. *J. Veg. Sci.* **19**, 875–884. 10.3170/2008-8-18462 (2008).

[28] Carrasco-Puga, G. et al. Revealing hidden plant diversity in arid environments. *Ecography* **44**(1), 98–111. 10.1111/ecog.05100 (2021).

[29] Venable, D. L., Flores-Martinez, A., Muller-Landau, H. C., Barron-Gafford, G. & Becerra, J. X. Seed dispersal of desert annuals. *Ecology* **89**(8), 2218–2227. 10.1890/07-0386.1 (2008). 18724732 10.1890/07-0386.1

[30] Shiferaw, W., Demissew, S. & Bekele, T. Ecology of soil seed banks: Implications for conservation and restoration of natural vegetation: A review. *Int. J. Biodivers. Conserv.* **10**(10), 380–393. 10.5897/IJBC2018.1226 (2018).

[31] Eskandari Dameneh, H. et al. Desertification of Iran in the early twenty-first century: assessment using climate and vegetation indices. *Sci. Rep.* **11**(1), 1–18. 10.1038/s41598-021-99636-8 (2021). 34654866 10.1038/s41598-021-99636-8 PMC8519952

[32] Jafari, R. & Abedi, M. Remote sensing-based biological and nonbiological indices for evaluating desertification in Iran: Image versus field indices. *Land. Degrad. Dev.* **32**(9), 2805–2822. 10.1002/ldr.3958 (2021).

[33] Emberger, L. Climate on a formula applicable in botanical geography. Comptes Rendus de l’Académie des Sciences 389–390 (1991).

[34] De Martonne, E. & Aerisme et índices d’aridite. *Comptes Rendus Acad. Sci.* **182**, 1395–1398 (1926).

[35] dos Santos, D. M., dos Santos, J. M. F. F., da Silva, K. A., de Araújo, V. K. R. & de Lima Araújo, E. Composition, species richness, and density of the germinable seed bank over 4 years in young and mature forests in Brazilian semiarid regions. *J. Arid Environ.* **129**, 93–101 (2016).

[36] Erfanzadeh, R., Kamali, P., Ghelichnia, H. & Pétillon, J. Effect of grazing removal on aboveground vegetation and soil seed bank composition in subalpine grasslands of northern Iran. *Plant. Ecol. Divers.* **9**(3), 309–320. 10.1080/17550874.2016.1221479 (2016).

[37] Mueller-Dombois, D. & Ellenberg, H. *Aims and methods of vegetation ecology* (Wiley, 1974). 10.2307/213332

[38] Mozaffarian, V. A. Flora of Ilam 4 (Farhange Moaser Publication, 2009).

[39] Raunkiaer, C. *The Life Forms of Plant and Statistical Plant Geography* 328 (Clarendon, 1934).

[40] Buzas, M. A. & Gibson, T. G. Species diversity: benthonic foraminifera in western North Atlantic. *Science* **163**(3862), 72–75. 10.1126/science.163.3862.72 (1969). 17780177 10.1126/science.163.3862.72

[41] Chao, A. & Jost, L. Coverage-based rarefaction and extrapolation: standardizing samples by completeness rather than size. *Ecology* **93**(12), 2533–2547. 10.1890/11-1952.1 (2012). 23431585 10.1890/11-1952.1

[42] Hsieh, T. C., Ma, K. & Chao, A. iNEXT: an R package for rarefaction and extrapolation of species diversity (H ill numbers). *Methods Ecol. Evol.* **7**(12), 1451–1456. 10.1111/2041-210X.12613 (2016).

[43] Wickham, H., Chang, W. & Wickham, M. H. Package ‘ggplot2’. *Create elegant data visualisations using Gramm. graphics Version*. **2**(1), 1–189 (2016).

[44] Hoyle, G. L. et al. Soil warming increases plant species richness but decreases germination from the alpine soil seed bank. *Glob. Change Biol.* **19**(5), 1549–1561. 10.1111/gcb.12135 (2013). 10.1111/gcb.12135 23505066

[45] Schneider, H. E. & Allen, E. B. Effects of elevated nitrogen and exotic plant invasion on soil seed bank composition in Joshua Tree National Park. *Plant Ecol.* **213**, 1277–1287. 10.1007/s11258-012-0085-6 (2012).

[46] Arruda, A. J. Seed ecology and grassland resilience: the case of campo rupestre (Doctoral dissertation, Université d’Avignon; Universidade federal de Minas Gerais. Facultade de educação (Brésil)) (2019).

[47] Omidi, M. et al. Evaluating the restoration potential of soil seed banks in degraded semiarid oak forests: Influence of canopy cover types and fire-related cues on seed germination. *For. Ecol. Manag.*10.1016/j.foreco.2022.120534 (2022).

[48] Zhang, D., Zhang, J., Yang, W., Wu, F. & Huang, Y. Plant and soil seed bank diversity across a range of ages of Eucalyptus grandis plantations afforested on arable lands. *Plant. soil.* **376**, 307–325. 10.1007/s11104-013-1954-z (2014).

[49] Zhao, Y., Li, M., Deng, J. & Wang, B. Afforestation affects soil seed banks by altering soil properties and understory plants on the eastern Loess Plateau, China. *Ecol. Ind.* **126**, 107670. 10.1016/j.ecolind.2021.107670 (2021).

[50] Dou, Y., Yang, Y., An, S. & Zhu, Z. Effects of different vegetation restoration measures on soil aggregate stability and erodibility on the Loess Plateau, China. *Catena* **185**, 104294. 10.1016/j.catena.2019.104294 (2020).

[51] Van Calster, H. et al. Long-term seed bank dynamics in a temperate forest under conversion from coppice‐with‐standards to high forest management. *Appl. Veg. Sci.* **11**(2), 251–260. 10.3170/2008-7-18405 (2008).

[52] Abtahi, M. Investigation of biodegradable polymer-cellulosic mulch persistence and effects on seed germination and establishment of desert. *Iran. J. Range Desert Res.* **26**(3), 517–530. 10.22092/ijrdr.2019.119986 (2019).

[53] Ghollasimod, S. & Memarian, H. Edaphically investigation of the Somaq habitat and its relationship with biological diversity (Case study: Bideskan habitat, Ferdows, Southeastern Khorasan, Iran). *Desert Ecosyst. Eng. J.* **7**(19), 17–32. 10.22052/deej.2018.7.19.11 (2018).

[54] Maestre, F. T. et al. Structure and functioning of dryland ecosystems in a changing world. *Annu. Rev. Ecol. Evol. Syst.* **47**(1), 215–237. 10.1146/annurev-ecolsys-121415-032311 (2016). 28239303 10.1146/annurev-ecolsys-121415-032311 PMC5321561

[55] Heydari, M., Pourbabaei, H., Esmaelzade, O., Pothier, D. & Salehi, A. Germination characteristics and diversity of soil seed banks and above-ground vegetation in disturbed and undisturbed oak forests. *For. Sci. Pract.* **15**, 286–301. 10.1007/s11632-013-0413-5 (2013).

[56] Rokich, D. P., Dixon, K. W., Sivasithamparam, K. & Meney, K. A. Smoke, mulch, and seed broadcasting effects on woodland restoration in Western Australia. *Restor. Ecol.* **10**(2), 185–194. 10.1046/j.1526-100X.2002.02040.x (2002).

[57] Valkó, O., Rádai, Z. & Deák, B. Hay transfer is a nature-based and sustainable solution for restoring grassland biodiversity. *J. Environ. Manage.* **311**, 114816. 10.1016/j.jenvman.2022.114816 (2022). 35248932 10.1016/j.jenvman.2022.114816

[58] Alrababah, M. A., Alhamad, M. A., Suwaileh, A. & Al-Gharaibeh, M. Biodiversity of semi‐arid Mediterranean grasslands: Impact of grazing and afforestation. *Appl. Veg. Sci.* **10**(2), 257–264. 10.1111/j.1654-109X.2007.tb00524.x (2007).

[59] Navarro, F. B., Jiménez, M. N., Gallego, E. & Ripoll, M. A. Short-term effects of overstory reduction and slash mulching on ground vegetation in a Mediterranean Aleppo pine woodland. *Eur. J. For. Res.* **129**(4), 689–696. 10.1007/s10342-010-0374-3 (2010).

[60] Shaw, N. et al. Seed use in the field: delivering seeds for restoration success. *Restor. Ecol.* **28**, S276–S285. 10.1111/rec.13210 (2020).

[61] Fehmi, J. S. & Kong, T. M. Effects of soil type, rainfall, straw mulch, and fertilizer on semiarid vegetation establishment, growth and diversity. *Ecol. Eng.* **44**, 70–77. 10.1016/j.ecoleng.2012.04.014 (2012).

[62] Egawa, C. & Tsuyuzaki, S. The effects of litter accumulation through succession on seed bank formation for small-and large‐seeded species. *J. Veg. Sci.* **24**(6), 1062–1073. 10.1111/jvs.12037 (2013).

[63] Farrell, C., Hobbs, R. J. & Colmer, T. D. Microsite and litter cover effects on seed banks vary with seed size and dispersal mechanisms: implications for revegetation of degraded saline land. *Plant Ecol.* **213**, 1145–1155. 10.1007/s11258-012-0072-y (2012).

