# Supplementary material for: Diversity and composition of vegetation and soil seed banks after sand dune restoration by oil mulching and plantations
Source: Sci Rep. 2025 Jan 25;15:3275. doi: 10.1038/s41598-024-83095-y (PMC11762977; doi:10.1038/s41598-024-83095-y)
Supplement: Supplementary file 1 — Supplementary Material 1 [file 41598_2024_83095_MOESM1_ESM.docx]

**Appendix**

Supplementary table 1. Plant composition, chorology, vegetative form, life form and soil seed bank by region. T= Iran-Turani, M = Mediterranean, Es= European Siberia, SS = Sahara-Sindian, Cosm = Cosmopolitan, Th= Therophytes, He= Hemicryptophytes, Cr= Cryptophytes, Ch= Chamophyte, Ph= Phanerophytes, A= Annual, B= Biennial, P = Perennials, F= Furb, G=Grass

| Species | Family | Life form | Chorology | Control | | Mulch 2017 | | Mulch 2019 | | Planted | |
| --- | --- | --- | --- | --- | --- | --- | --- | --- | --- | --- | --- |
|  |  |  |  | soil seed bank | Ground cover | soil seed bank | Ground cover | soil seed bank | Ground cover | soil seed bank | Ground cover |
| *Anagallis arvensis* | Primulaceae | Th | IT-Med |  |  |  |  |  |  | * | * |
| *Artemisia sieberi* | Asteraceae | He | IT-Eur-sib |  | * |  |  |  | * |  |  |
| *Asphodelus* *tenuifolius* | Liliaceae | Th | IT-med |  | * |  | * |  | * |  | * |
| *Astragalus* *asterias* | Fabaceae | Th | IT-med |  |  |  |  |  |  |  | * |
| *Bromus scoparius* | Poaceae | Th | IT-med |  |  |  |  |  |  | * | * |
| *Bromus tectorum* | Poaceae | Th | IT-med |  | * |  |  |  |  |  | * |
| *Calendula persica* | Asteraceae | Th | IT-med |  |  |  |  |  |  | * |  |
| *Carduus arabicus* | Asteraceae | Th | IT |  | * |  |  |  | * |  |  |
| *Carex stenophylla* | Cyperaceae | Th | IT-SS |  | * | * | * | * |  | * | * |
| *Carthmus oxyacantha* | Asteraceae | Th | IT |  |  |  | * |  |  |  |  |
| *Centaurea bruguierana* | Asteraceae | Th | IT-SS |  |  |  |  |  | * |  |  |
| *Chrozophora tinctoria* | Euphorbiaceae | Th | IT-SS |  | * |  |  |  |  |  |  |
| *Cornulaca* *aucheri* | Chenopodiaceae | He | IT-SS |  | * |  | * | * | * | * | * |
| *Convolvulus* *oxyphyllus* | Convolvulaceae | He | IT-SS |  | * |  |  |  | * |  |  |
| *Cymbolaena griffithii* | Asteraceae | Th | IT-Med |  | * |  |  |  |  |  |  |
| *Cyperus eremicus* | Cyperaceae | He | IT-Med |  | * |  |  |  | * |  |  |
| *Diplotaxis harra* | Cruciferae | Th | IT-SS |  | * |  | * |  |  |  | * |
| *Emex spinosus* | Polygonaceae | Th | IT-SS |  |  | * | * |  |  |  | * |
| *Erodium moschatum* | Geraniaceae | Th | IT-Med |  | * |  |  |  | * |  |  |
| *Hedypnois rhagadioloides* | Asteraceae | Th | IT-Mes-Ss |  |  |  |  |  |  |  | * |
| *Heliotropium ramosissimum* | Boraginaceae | He | IT-SS |  |  |  | * |  |  |  | * |
| *Hordeum glaucum* | Poaceae | Th | Cos-IT |  |  |  |  |  |  |  | * |
| *Lolium rigidum* | Poaceae | Th | IT-Cos-Ms |  |  |  | * |  |  |  |  |
| *Lophochloa phleodies* | Poaceae | Th | IT-SS-Cos | * | * |  | * |  |  | * | * |
| *Malva parviflora* | [Malvaceae](https://en.wikipedia.org/wiki/Malvaceae) | Th | IT-SS |  |  | * | * |  |  |  | * |
| *Medicago radiata* | Fabaceae | Th | Cos-IT-SS-Med |  |  | * |  | * |  |  |  |
| *Neurada procumbens* | Rosaceae | Th | SS |  | * |  | * |  | * |  | * |
| *Plantago amplexicaulis* | Plantaginaceae | Th | IT-SS | * | * | * | * | * | * | * | * |
| *Senecio glaucus* | Asteraceae | Th | IT-SS |  |  |  | * |  |  |  |  |
| *Stipa capensis* | Poaceae | Th | IT-SS |  |  |  | * |  |  | * | * |
| *Stipagrostis plumosa* | Poaceae | He | IT-Cos-SS |  | * |  |  |  |  |  |  |
| *Taraxacum montanum* | Asteraceae | Th | IT- Med –SS |  |  |  | * |  |  |  | * |
| *Trifolium lappaceum* | Fabaceae | Th | IT-Cos-Med |  | * |  | * |  | * |  | * |
| *Trifolium tomentosum* | Fabaceae | Th | IT-Cos-Med |  |  |  | * |  | * |  | * |
| *Trifolium resupinatum* | Fabaceae | Th | IT-Cos-Med |  |  |  |  |  |  |  | * |
| *Trigonella anguinea* | Fabaceae | Th | IT-SS |  | * |  | * |  | * |  | * |

Supplementary table 2. List and geographical distribution of plants in the first ecological group. Ground cover in the control area

| **Life form** | **Family** | **Species name** |
| --- | --- | --- |
| He | Asteraceae | *Artemisia sieberi* |
| Th | Liliaceae | *Asphodelus* *tenuifolius* |
| Th | Poaceae | *Bromus tectorum* |
| Th | Asteraceae | *Carduus arabicus* |
| He | Cyperaceae | *Carex stenophylla* |
| Th | Euphorbiaceae | *Chrozophora tinctoria* |
| He | Chenopodiaceae | *Cornulaca* *aucheri* |
| He | Convolvulaceae | *Convolvulus* *oxyphyllus* |
| Th | Asteraceae | *Cymbolaena griffithii* |
| He | Cyperaceae | *Cyperus eremicus* |
| Th | Cruciferae | *Diplotaxis* *harra* |
| Th | Geraniaceae | *Erodium moschatum* |
| Th | Poaceae | *Lophochloa phleodies* |
| Th | Rosaceae | *Neurada procumbens* |
| Th | Plantaginaceae | *Plantago amplexicaulis* |
| Th | Poaceae | *Stipa capensis* |
| He | Poaceae | *Stipagrostis plumosa* |
| Th | Fabaceae | *Trifolium lappaceum* |
| Th | Fabaceae | *Trigonella anguinea* |

Supplementary table 3. List and geographical distribution of plants in the second ecological group. Ground cover in the 2017 mulch area

| **Life form** | **Family** | **Species name** |
| --- | --- | --- |
| Th | Liliaceae | *Asphodelus* *tenuifolius* |
| **He** | Cyperaceae | *Carex stenophylla* |
| Th | Asteraceae | *Carthmus oxyacantha* |
| Th | Asteraceae | *Carduus arabicus* |
| Th | Cruciferae | *Diplotaxis* *harra* |
| Th | Polygonaceae | *Emex spinosus* |
| **He** | Boraginaceae | *Heliotropium ramosissimum* |
| Th | Poaceae | *Lolium rigidum* |
| Th | Poaceae | *Lophochloa phleodies* |
| Th | [Malvaceae](https://en.wikipedia.org/wiki/Malvaceae) | *Malva parviflora* |
| Th | Rosaceae | *Neurada procumbens* |
| Th | Plantaginaceae | *Plantago amplexicaulis* |
| Th | Asteraceae | *Senecio glaucus* |
| Th | Poaceae | *Stipa capensis* |
| Th | Asteraceae | *Taraxacum montanum* |
| Th | Fabaceae | *Trifolium lappaceum* |
| Th | Fabaceae | *Trifolium tomentosum* |
| Th | Fabaceae | *Trigonella anguinea* |

Supplementary table 4. List and geographical distribution of plants in the third ecological group. Ground cover in the 2019 mulch area

| **Life form** | **Family** | **Species name** |
| --- | --- | --- |
| He | Asteraceae | *Artemisia sIieberi* |
| Th | Liliaceae | *Asphodelus* *tenuifolius* |
| Th | Asteraceae | *Carduus arabicus* |
| Th | Asteraceae | *Centaurea bruguierana* |
| He | Chenopodiaceae | *Cornulaca* *aucheri* |
| He | Convolvulaceae | *Convolvulus* *oxyphyllus* |
| He | Cyperaceae | *Cyperus eremicus* |
| Th | Geraniaceae | *Erodium moschatum* |
| Th | Rosaceae | *Neurada procumbens* |
| Th | Plantaginaceae | *Plantago amplexicaulis* |
| Th | Fabaceae | *Trifolium lappaceum* |
| Th | Fabaceae | *Trifolium tomentosum* |
| Th | Fabaceae | *Trigonella anguinea* |

Supplementary table 5. List and geographical distribution of plants in the fourth ecological group. Ground cover in the planted area

| **Life form** | **Family** | **Species name** |
| --- | --- | --- |
| Th | Primulaceae | *Anagallis arvensis* |
| Th | Liliaceae | *Asphodelus* *tenuifolius* |
| Th | Fabaceae | *Astragalus asterias* |
| Th | Poaceae | *Bromus scoparius* |
| Th | Poaceae | *Bromus tectorum* |
| **He** | Cyperaceae | *Carex stenophylla* |
| He | Chenopodiaceae | *Cornulaca* *aucheri* |
| Th | Cruciferae | *Diplotaxis* *harra* |
| Th | Polygonaceae | *Emex spinosus* |
| Th | Asteraceae | *Hedypnois rhagadioloides* |
| **He** | Boraginaceae | *Heliotropium ramosissimum* |
| Th | Poaceae | *Hordeum glaucum* |
| Th | Poaceae | *Lophochloa phleodies* |
| Th | [Malvaceae](https://en.wikipedia.org/wiki/Malvaceae) | *Malva parviflora* |
| Th | Rosaceae | *Neurada procumbens* |
| Th | Plantaginaceae | *Plantago amplexicaulis* |
| Th | Poaceae | *Stipa capensis* |
| Th | Asteraceae | *Taraxacum montanum* |
| Th | Fabaceae | *Trifolium lappaceum* |
| Th | Fabaceae | *Trifolium tomentosum* |
| Th | Fabaceae | *Trifolium resupinatum* |
| Th | Fabaceae | *Trigonella anguinea* |
